# Supplementary material for: A Likelihood Approach for Real-Time Calibration of Stochastic Compartmental Epidemic Models
Source: PLoS Comput Biol. 2017 Jan 17;13(1):e1005257. doi: 10.1371/journal.pcbi.1005257 (PMC5240920; doi:10.1371/journal.pcbi.1005257)
Supplement: S1 File — (TAR.GZ) [file pcbi.1005257.s014.tar.gz › HSPH_Online-SI-Revision/IPoi09/n1-extreme/n1-extreme_BENCH2_table4.pdf]

| | | | | | | | | | | | | | | | | | | | | | | | | | | | | | | |
|---|---|---|---|---|---|---|---|---|---|---|---|---|---|---|---|---|---|---|---|---|---|---|---|---|---|---|---|---|---|---|
| 1 | newly 3 weeks spec | 16. | 24.8689 | {11., 39.} | 67.3156 | 0.193264 | newly 3 weeks cumul | 86. | 104.984 | {57., 153.} | 44.7198 | 0.15485 | newly 5 weeks spec | 10. | 12.2951 | {3., 22.} | 67.541 | 0.270595 | newly 5 weeks cumul | 112. | 134.934 | {73., 192.} | 45.9163 | 0.162592 | newly 56 weeks cumul | 131. | 160.885 | {84., 239.} | 47.8914 | 0.165995 |
| 2 | newly 3 weeks spec | 15. | 28.4918 | {14., 45.} | 94.0984 | 0.237783 | newly 3 weeks cumul | 66. | 107.295 | {62., 184.} | 66.6418 | 0.190107 | newly 5 weeks spec | 14. | 18.0164 | {7., 29.} | 57.4941 | 0.200109 | newly 5 weeks cumul | 87. | 148.721 | {87., 247.} | 74.8634 | 0.204358 | newly 52 weeks cumul | 117. | 211.639 | {106., 336.} | 85.2599 | 0.219534 |
| 3 | newly 3 weeks spec | 43. | 41.9836 | {23., 65.} | 33.0156 | 0.152631 | newly 3 weeks cumul | 138. | 150.016 | {94., 208.} | 27.2155 | 0.111034 | newly 5 weeks spec | 26. | 27.6885 | {13., 41.} | 43.947 | 0.174725 | newly 5 weeks cumul | 184. | 212.262 | {133., 306.} | 31.9316 | 0.120995 | newly 54 weeks cumul | 247. | 314. | {178., 438.} | 43.1473 | 0.145869 |
| 4 | newly 3 weeks spec | 35. | 35.4308 | {16., 52.} | 36.5714 | 0.163809 | newly 3 weeks cumul | 105. | 128.492 | {69., 195.} | 40.1612 | 0.149235 | newly 5 weeks spec | 26. | 22.8923 | {9., 34.} | 47.929 | 0.23984 | newly 5 weeks cumul | 155. | 180. | {91., 292.} | 39.196 | 0.150246 | newly 53 weeks cumul | 252. | 260.108 | {116., 439.} | 39.7985 | 0.168642 |
| 5 | newly 3 weeks spec | 18. | 0. | {0., 0.} | 100. | 2.25527 | newly 3 weeks cumul | 78. | 0. | {0., 0.} | 100. | 2.89209 | newly 5 weeks spec | 14. | 0. | {0., 0.} | 100. | 2.14613 | newly 5 weeks cumul | 116. | 0. | {0., 0.} | 100. | 3.06446 | newly 46 weeks cumul | 161. | 0. | {0., 0.} | 100. | 3.20683 |
| 6 | newly 3 weeks spec | 29. | 29.8615 | {14., 50.} | 42.6525 | 0.186019 | newly 3 weeks cumul | 76. | 110.785 | {59., 187.} | 59.2105 | 0.186667 | newly 5 weeks spec | 16. | 19.5077 | {7., 36.} | 58.6538 | 0.221454 | newly 5 weeks cumul | 103. | 155. | {75., 258.} | 63.062 | 0.191947 | newly 51 weeks cumul | 158. | 223.815 | {99., 420.} | 61.6943 | 0.194994 |
| 7 | newly 3 weeks spec | 23. | 22.9857 | {10., 34.} | 42.0497 | 0.191052 | newly 3 weeks cumul | 107. | 91.5 | {44., 138.} | 35.7944 | 0.185959 | newly 5 weeks spec | 18. | 12.3286 | {6., 20.} | 45.9524 | 0.282465 | newly 5 weeks cumul | 144. | 120.471 | {57., 184.} | 36.0813 | 0.189907 | newly 55 weeks cumul | 190. | 150.557 | {64., 241.} | 38.4135 | 0.210755 |
| 8 | newly 3 weeks spec | 21. | 16.8983 | {5., 33.} | 58.4342 | 0.334564 | newly 3 weeks cumul | 65. | 65.5593 | {23., 125.} | 53.0639 | 0.241593 | newly 5 weeks spec | 12. | 11.1356 | {3., 24.} | 69.6328 | 0.371311 | newly 5 weeks cumul | 85. | 91.4915 | {31., 171.} | 57.8465 | 0.243905 | newly 49 weeks cumul | 108. | 139.339 | {41., 259.} | 71.5474 | 0.249829 |
| 9 | newly 3 weeks spec | 14. | 21.274 | {7., 46.} | 78.3757 | 0.237 | newly 3 weeks cumul | 73. | 92.9863 | {36., 201.} | 59.2419 | 0.204475 | newly 5 weeks spec | 7. | 11.2603 | {2., 26.} | 100.783 | 0.354947 | newly 5 weeks cumul | 94. | 119.658 | {49., 266.} | 60.9589 | 0.211232 | newly 56 weeks cumul | 112. | 144.945 | {55., 317.} | 63.8087 | 0.217663 |
| 10 | newly 3 weeks spec | 36. | 21.9661 | {7., 42.} | 53.7665 | 0.371163 | newly 3 weeks cumul | 109. | 80.0508 | {30., 150.} | 45.3118 | 0.271443 | newly 5 weeks spec | 14. | 14.7966 | {3., 31.} | 66.4649 | 0.295172 | newly 5 weeks cumul | 147. | 111.746 | {40., 209.} | 48.2417 | 0.280346 | newly 49 weeks cumul | 210. | 170.729 | {51., 312.} | 54.9233 | 0.300557 |
| 11 | newly 3 weeks spec | 14. | 14.4844 | {5., 27.} | 58.3705 | 0.257299 | newly 3 weeks cumul | 74. | 62.9063 | {26., 119.} | 47.7196 | 0.244042 | newly 5 weeks spec | 6. | 6.625 | {2., 13.} | 65.625 | 0.273988 | newly 5 weeks cumul | 95. | 79.5625 | {35., 154.} | 48.125 | 0.249743 | newly 55 weeks cumul | 108. | 96.0313 | {40., 192.} | 49.9132 | 0.244702 |
| 12 | newly 3 weeks spec | 26. | 35.9661 | {17., 56.} | 57.1056 | 0.191553 | newly 3 weeks cumul | 91. | 142.186 | {80., 217.} | 63.3638 | 0.192868 | newly 5 weeks spec | 9. | 19.3729 | {8., 34.} | 119.774 | 0.289414 | newly 5 weeks cumul | 114. | 187.949 | {103., 277.} | 70.5471 | 0.204817 | newly 56 weeks cumul | 127. | 238.034 | {121., 354.} | 90.6313 | 0.242002 |
| 13 | newly 3 weeks spec | 35. | 26.5902 | {11., 48.} | 44.0749 | 0.249413 | newly 3 weeks cumul | 94. | 94.1148 | {41., 159.} | 36.2923 | 0.164355 | newly 5 weeks spec | 33. | 19.7705 | {7., 41.} | 54.4958 | 0.360739 | newly 5 weeks cumul | 163. | 136.082 | {58., 254.} | 40.0885 | 0.210217 | newly 49 weeks cumul | 326. | 215.361 | {91., 434.} | 50.8348 | 0.309255 |
| 14 | newly 3 weeks spec | 7. | 18. | {7., 40.} | 160.19 | 0.347905 | newly 3 weeks cumul | 41. | 72.48 | {33., 146.} | 84.4553 | 0.229553 | newly 5 weeks spec | 2. | 10.4667 | {4., 19.} | 427.333 | 0.653966 | newly 5 weeks cumul | 46. | 96.2667 | {43., 186.} | 113.275 | 0.282643 | newly 53 weeks cumul | 53. | 125.907 | {59., 242.} | 140.277 | 0.327259 |
| 15 | newly 3 weeks spec | 12. | 26.1607 | {9., 54.} | 126.042 | 0.307166 | newly 3 weeks cumul | 65. | 93.75 | {38., 165.} | 58.4066 | 0.191856 | newly 5 weeks spec | 12. | 18.9107 | {7., 44.} | 80.2083 | 0.231373 | newly 5 weeks cumul | 89. | 135. | {55., 260.} | 65.6501 | 0.203677 | newly 48 weeks cumul | 106. | 213.625 | {88., 434.} | 110.024 | 0.274723 |
| 16 | newly 3 weeks spec | 31. | 14.7895 | {6., 24.} | 57.1307 | 0.413509 | newly 3 weeks cumul | 81. | 62.9079 | {31., 102.} | 39.7173 | 0.221307 | newly 5 weeks spec | 20. | 7.85526 | {3., 15.} | 60.9868 | 0.509948 | newly 5 weeks cumul | 118. | 81.0789 | {39., 132.} | 42.6182 | 0.254706 | newly 55 weeks cumul | 180. | 100.645 | {46., 169.} | 49.9781 | 0.328949 |
| 17 | newly 3 weeks spec | 33. | 37.3571 | {15., 72.} | 53.9827 | 0.222619 | newly 3 weeks cumul | 111. | 132.786 | {63., 227.} | 48.816 | 0.187775 | newly 5 weeks spec | 26. | 24.2286 | {7., 46.} | 52.4176 | 0.264842 | newly 5 weeks cumul | 160. | 186.729 | {88., 329.} | 49.8482 | 0.1963 | newly 52 weeks cumul | 266. | 279.129 | {120., 531.} | 49.4898 | 0.214723 |
| 18 | newly 3 weeks spec | 26. | 23.1884 | {10., 42.} | 40.3567 | 0.206012 | newly 3 weeks cumul | 118. | 91.3768 | {48., 173.} | 40.985 | 0.222655 | newly 5 weeks spec | 13. | 13.9275 | {4., 34.} | 55.2954 | 0.244696 | newly 5 weeks cumul | 153. | 122.928 | {64., 242.} | 41.7448 | 0.221558 | newly 55 weeks cumul | 200. | 161.145 | {79., 315.} | 43.529 | 0.231342 |
| 19 | newly 3 weeks spec | 32. | 22.7183 | {8., 38.} | 48.0194 | 0.290469 | newly 3 weeks cumul | 144. | 90.7465 | {40., 138.} | 46.9777 | 0.296575 | newly 5 weeks spec | 11. | 11.8451 | {4., 22.} | 53.7772 | 0.235055 | newly 5 weeks cumul | 177. | 118.268 | {53., 186.} | 46.1367 | 0.280941 | newly 56 weeks cumul | 231. | 147.549 | {63., 249.} | 48.5397 | 0.301525 |
| 20 | newly 3 weeks spec | 8. | 19.1311 | {7., 34.} | 146.107 | 0.324686 | newly 3 weeks cumul | 57. | 73.3934 | {32., 125.} | 55.22 | 0.188797 | newly 5 weeks spec | 5. | 12.1639 | {3., 22.} | 158.361 | 0.353917 | newly 5 weeks cumul | 69. | 102.23 | {45., 175.} | 66.928 | 0.198513 | newly 49 weeks cumul | 94. | 146.131 | {61., 257.} | 72.9334 | 0.204444 |
| 21 | newly 3 weeks spec | 40. | 40.4923 | {19., 67.} | 37.3077 | 0.170205 | newly 3 weeks cumul | 123. | 141.477 | {84., 206.} | 34.8343 | 0.137973 | newly 5 weeks spec | 31. | 28.0154 | {11., 49.} | 41.1911 | 0.210866 | newly 5 weeks cumul | 179. | 202.154 | {109., 310.} | 35.8659 | 0.144427 | newly 52 weeks cumul | 267. | 319.123 | {140., 620.} | 46.2345 | 0.175195 |
| 22 | newly 3 weeks spec | 22. | 14.013 | {4., 30.} | 51.6529 | 0.351493 | newly 3 weeks cumul | 81. | 56.5584 | {23., 118.} | 48.7093 | 0.293473 | newly 5 weeks spec | 11. | 8.57143 | {2., 19.} | 56.7887 | 0.333309 | newly 5 weeks cumul | 100. | 75.7273 | {30., 146.} | 48.8701 | 0.277337 | newly 53 weeks cumul | 148. | 98.1429 | {37., 192.} | 51.1144 | 0.317733 |
| 23 | newly 3 weeks spec | 17. | 28.3562 | {15., 42.} | 72.4416 | 0.2047 | newly 3 weeks cumul | 88. | 113.959 | {61., 174.} | 44.8786 | 0.15345 | newly 5 weeks spec | 9. | 16.4384 | {6., 39.} | 95.7382 | 0.252931 | newly 5 weeks cumul | 105. | 151.74 | {81., 232.} | 54.8989 | 0.170748 | newly 55 weeks cumul | 155. | 198.438 | {101., 303.} | 46.6726 | 0.159473 |
| 24 | newly 3 weeks spec | 24. | 24.8689 | {11., 39.} | 86.2998 | 0.231386 | newly 3 weeks cumul | 65. | 104.984 | {57., 153.} | 68.2219 | 0.190347 | newly 5 weeks spec | 7. | 12.2951 | {3., 22.} | 103.279 | 0.297132 | newly 5 weeks cumul | 79. | 134.934 | {73., 192.} | 76.7794 | 0.208014 | newly 56 weeks cumul | 97. | 160.885 | {84., 239.} | 73.7367 | 0.201641 |
| 25 | newly 3 weeks spec | 37. | 32.0896 | {14., 56.} | 40.7826 | 0.21163 | newly 3 weeks cumul | 118. | 115.463 | {57., 194.} | 35.9474 | 0.165356 | newly 5 weeks spec | 14. | 20.209 | {7., 35.} | 70.3625 | 0.227092 | newly 5 weeks cumul | 153. | 161.194 | {81., 274.} | 40.1619 | 0.170495 | newly 53 weeks cumul | 253. | 231.433 | {112., 397.} | 43.7555 | 0.209055 |
| 26 | newly 3 weeks spec | 7. | 35.9768 | {18., 55.} | 414.066 | 0.652922 | newly 3 weeks cumul | 46. | 138.462 | {76., 218.} | 201.003 | 0.440675 | newly 5 weeks spec | 5. | 22.2462 | {8., 35.} | 346.769 | 0.56773 | newly 5 weeks cumul | 56. | 189.077 | {97., 305.} | 237.637 | 0.48431 | newly 55 weeks cumul | 70. | 253.662 | {128., 403.} | 262.374 | 0.505206 |
| 27 | newly 3 weeks spec | 41. | 19.0161 | {8., 34.} | 59.1267 | 0.445149 | newly 3 weeks cumul | 120. | 73.5968 | {32., 125.} | 48.8844 | 0.312211 | newly 5 weeks spec | 16. | 12.3226 | {4., 22.} | 52.621 | 0.317857 | newly 5 weeks cumul | 166. | 102.532 | {45., 175.} | 50.3012 | 0.319807 | newly 50 weeks cumul | 260. | 146.71 | {61., 257.} | 53.9826 | 0.359214 |
| 28 | newly 3 weeks spec | 13. | 31.1765 | {15., 67.} | 142.76 | 0.325431 | newly 3 weeks cumul | 77. | 123. | {61., 246.} | 68.9076 | 0.19695 | newly 5 weeks spec | 17. | 18.5294 | {6., 40.} | 59.6886 | 0.257415 | newly 5 weeks cumul | 109. | 165.662 | {81., 319.} | 64.7464 | 0.189913 | newly 54 weeks cumul | 162. | 220.868 | {95., 435.} | 58.3969 | 0.188678 |
| 29 | newly 3 weeks spec | 7. | 29.8714 | {12., 58.} | 326.735 | 0.554696 | newly 3 weeks cumul | 71. | 112.2 | {50., 200.} | 74.004 | 0.220233 | newly 5 weeks spec | 8. | 20.5857 | {6., 39.} | 167.321 | 0.356771 | newly 5 weeks cumul | 91. | 157.486 | {69., 295.} | 85.2433 | 0.233394 | newly 51 weeks cumul | 100. | 231.371 | {100., 458.} | 134.257 | 0.299461 |
| 30 | newly 3 weeks spec | 10. | 19.9655 | {3., 24.} | 76.2069 | 0.34623 | newly 3 weeks cumul | 42. | 42.7414 | {12., 85.} | 64.8194 | 0.29255 | newly 5 weeks spec | 6. | 7.81034 | {1., 15.} | 85.9195 | 0.383917 | newly 5 weeks cumul | 53. | 61.1207 | {17., 116.} | 69.6487 | 0.276963 | newly 46 weeks cumul | 78. | 90.2586 | {24., 168.} | 75.3537 | 0.301044 |
| 31 | newly 3 weeks spec | 10. | 14.4615 | {5., 25.} | 67.0769 | 0.2142 | newly 3 weeks cumul | 87. | 61.5692 | {28., 107.} | 42.847 | 0.257639 | newly 5 weeks spec | 4. | 7.69231 | {2., 16.} | 116.923 | 0.342019 | newly 5 weeks cumul | 99. | 78. | {35., 146.} | 42.6729 | 0.232751 | newly 55 weeks cumul | 122. | 99.8308 | {42., 186.} | 43.0895 | 0.232051 |
| 32 | newly 3 weeks spec | 6. | 33. | {15., 56.} | 450. | 0.677796 | newly 3 weeks cumul | 62. | 133.836 | {74., 215.} | 116.249 | 0.299751 | newly 5 weeks spec | 6. | 18.806 | {8., 36.} | 213.93 | 0.424654 | newly 5 weeks cumul | 78. | 178.507 | {98., 276.} | 128.856 | 0.320024 | newly 55 weeks cumul | 82. | 230.448 | {120., 357.} | 181.034 | 0.403407 |
| 33 | newly 3 weeks spec | 35. | 31.3898 | {11., 58.} | 43.2446 | 0.227424 | newly 3 weeks cumul | 123. | 109.169 | {42., 176.} | 40.1819 | 0.209511 | newly 5 weeks spec | 29. | 21.9661 | {7., 42.} | 53.0099 | 0.317888 | newly 5 weeks cumul | 183. | 157.831 | {59., 270.} | 41.8172 | 0.222505 | newly 51 weeks cumul | 278. | 248.508 | {84., 447.} | 49.9085 | 0.254346 |
| 34 | newly 3 weeks spec | 27. | 31.1688 | {16., 61.} | 46.0317 | 0.17324 | newly 3 weeks cumul | 98. | 123.377 | {61., 237.} | 46.8328 | 0.163874 | newly 5 weeks spec | 12. | 18.5584 | {7., 40.} | 74.5671 | 0.222118 | newly 5 weeks cumul | 137. | 165.584 | {82., 319.} | 45.4925 | 0.164688 | newly 54 weeks cumul | 165. | 220.052 | {101., 429.} | 53.7348 | 0.17755 |
| 35 | newly 3 weeks spec | 25. | 24.7733 | {13., 41.} | 36.4267 | 0.165706 | newly 3 weeks cumul | 103. | 100.293 | {60., 146.} | 31.2362 | 0.139927 | newly 5 weeks spec | 20. | 14.2133 | {6., 26.} | 44. | 0.256662 | newly 5 weeks cumul | 144. | 134.04 | {78., 201.} | 31.6389 | 0.147217 | newly 55 weeks cumul | 192. | 173.107 | {95., 262.} | 33.3264 | 0.160099 |
| 36 | newly 3 weeks spec | 28. | 28.4918 | {14., 45.} | 42.3888 | 0.176743 | newly 3 weeks cumul | 103. | 107.295 | {62., 184.} | 36.2566 | 0.149318 | newly 5 weeks spec | 9. | 18.0164 | {7., 29.} | 111.475 | 0.278546 | newly 5 weeks cumul | 131. | 148.721 | {87., 247.} | 40.3829 | 0.149993 | newly 52 weeks cumul | 175. | 211.639 | {106., 336.} | 46.9789 | 0.161953 |
| 37 | newly 3 weeks spec | 25. | 22.4821 | {8., 51.} | 47.2143 | 0.241161 | newly 3 weeks cumul | 95. | 80.4643 | {31., 158.} | 41.2782 | 0.216533 | newly 5 weeks spec | 12. | 15.5893 | {3., 35.} | 74.5536 | 0.292391 | newly 5 weeks cumul | 128. | 114.821 | {47., 239.} | 44.7545 | 0.222112 | newly 47 weeks cumul | 171. | 177.429 | {67., 362.} | 51.9006 | 0.221253 |
| 38 | newly 3 weeks spec | 18. | 23.4098 | {10., 37.} | 57.9235 | 0.183892 | newly 3 weeks cumul | 76. | 87.4754 | {46., 136.} | 42.1915 | 0.15121 | newly 5 weeks spec | 9. | 15.2787 | {6., 25.} | 87.6138 | 0.23466 | newly 5 weeks cumul | 96. | 120.77 | {59., 212.} | 47.6947 | 0.157117 | newly 52 weeks cumul | 127. | 168.41 | {80., 276.} | 53.9822 | 0.167381 |
| 39 | newly 3 weeks spec | 23. | 24.7097 | {10., 46.} | 52.5947 | 0.218862 | newly 3 weeks cumul | 103. | 98.7742 | {51., 184.} | 42.1234 | 0.188438 | newly 5 weeks spec | 6. | 15.0968 | {6., 30.} | 155.914 | 0.322878 | newly 5 weeks cumul | 136. | 132.5 | {66., 244.} | 44.509 | 0.19356 | newly 55 weeks cumul | 164. | 176.274 | {85., 319.} | 48.5543 | 0.189587 |
| 40 | newly 3 weeks spec | 14. | 16.7391 | {6., 39.} | 54.1408 | 0.204098 | newly 3 weeks cumul | 66. | 67.8116 | {33., 129.} | 43.1489 | 0.18483 | newly 5 weeks spec | 15. | 9.49275 | {3., 19.} | 56.4251 | 0.38865 | newly 5 weeks cumul | 96. | 89.971 | {40., 171.} | 42.9348 | 0.20021 | newly 53 weeks cumul | 143. | 117.667 | {49., 218.} | 44.4512 | 0.23269 |
| 41 | newly 3 weeks spec | 19. | 26.2321 | {10., 54.} | 59.6805 | 0.194839 | newly 3 weeks cumul | 71. | 93.875 | {38., 165.} | 49.3712 | 0.172609 | newly 5 weeks spec | 8. | 18.7679 | {6., 44.} | 143.973 | 0.317869 | newly 5 weeks cumul | 101. | 135.125 | {58., 260.} | 54.4222 | 0.178093 | newly 48 weeks cumul | 154. | 213.321 | {88., 434.} | 60.1577 | 0.192268 |
| 42 | newly 3 weeks spec | 21. | 17.6557 | {7., 30.} | 43.4036 | 0.234765 | newly 3 weeks cumul | 118. | 75.7049 | {37., 107.} | 47.9578 | 0.289344 | newly 5 weeks spec | 14. | 7.54098 | {2., 13.} | 55.5035 | 0.423488 | newly 5 weeks cumul | 148. | 95.541 | {49., 142.} | 48.0284 | 0.290205 | newly 56 weeks cumul | 159. | 113.951 | {57., 173.} | 45.3449 | 0.260139 |
| 43 | newly 3 weeks spec | 41. | 40.4474 | {18., 74.} | 37.484 | 0.169947 | newly 3 weeks cumul | 137. | 155.276 | {81., 275.} | 36.0449 | 0.140381 | newly 5 weeks spec | 20. | 24.1579 | {11., 42.} | 48.2895 | 0.181195 | newly 5 weeks cumul | 181. | 210.105 | {108., 394.} | 38.2233 | 0.144092 | newly 55 weeks cumul | 229. | 282.711 | {139., 540.} | 44.2657 | 0.157072 |
| 44 | newly 3 weeks spec | 21. | 34.2581 | {16., 51.} | 70.5069 | 0.208686 | newly 3 weeks cumul | 78. | 126.145 | {83., 178.} | 64.7022 | 0.197248 | newly 5 weeks spec | 11. | 22.3548 | {10., 36.} | 109.971 | 0.263783 | newly 5 weeks cumul | 103. | 176.145 | {108., 253.} | 73.301 | 0.211274 | newly 53 weeks cumul | 147. | 253.468 | {140., 353.} | 76.2892 | 0.209908 |
| 45 | newly 3 weeks spec | 21. | 30.1642 | {11., 65.} | 51.2082 | 0.304572 | newly 3 weeks cumul | 127. | 121.701 | {55., 255.} | 47.7729 | 0.21794 | newly 5 weeks spec | 16. | 14.8657 | {3., 34.} | 57.4627 | 0.308231 | newly 5 weeks cumul | 166. | 157.284 | {74., 325.} | 48.984 | 0.227082 | newly 56 weeks cumul | 228. | 191.672 | {83., 394.} | 50.8903 | 0.262689 |
| 46 | newly 3 weeks spec | 28. | 23.4098 | {10., 37.} | 49.5316 | 0.247761 | newly 3 weeks cumul | 111. | 87.4754 | {46., 136.} | 42.6377 | 0.220155 | newly 5 weeks spec | 15. | 15.2787 | {6., 25.} | 53.6612 | 0.234707 | newly 5 weeks cumul | 142. | 120.77 | {59., 212.} | 42.496 | 0.207177 | newly 53 weeks cumul | 196. | 168.41 | {80., 276.} | 44.9565 | 0.216218 |
| 47 | newly 3 weeks spec | 37. | 39.9859 | {20., 66.} | 40.9593 | 0.168844 | newly 3 weeks cumul | 141. | 144.718 | {81., 235.} | 31.3455 | 0.135232 | newly 5 weeks spec | 23. | 26.2113 | {12., 42.} | 45.4378 | 0.166993 | newly 5 weeks cumul | 188. | 203.958 | {114., 341.} | 34.8592 | 0.140233 | newly 54 weeks cumul | 283. | 290.549 | {156., 497.} | 38.3616 | 0.160842 |
| 48 | newly 3 weeks spec | 34. | 23.8 | {12., 38.} | 41.2157 | 0.243862 | newly 3 weeks cumul | 113. | 96.72 | {59., 164.} | 33.3097 | 0.166737 | newly 5 weeks spec | 18. | 14.04 | {6., 26.} | 45.2593 | 0.260741 | newly 5 weeks cumul | 163. | 128.933 | {77., 215.} | 35.9509 | 0.191048 | newly 54 weeks cumul | 230. | 173.867 | {100., 291.} | 38.771 | 0.212728 |
| 49 | newly 3 weeks spec | 30. | 17.2787 | {7., 30.} | 51.3661 | 0.348567 | newly 3 weeks cumul | 104. | 73.4426 | {35., 107.} | 45.9962 | 0.264379 | newly 5 weeks spec | 16. | 7.22951 | {2., 11.} | 61.5779 | 0.501752 | newly 5 weeks cumul | 140. | 92.5246 | {44., 142.} | 48.3841 | 0.289896 | newly 56 weeks cumul | 177. | 109.574 | {55., 173.} | 51.042 | 0.318439 |
| 50 | newly 3 weeks spec | 29. | 6.54667 | {2., 13.} | 78.1609 | 0.794106 | newly 3 weeks cumul | 85. | 26.4933 | {9., 56.} | 72.0941 | 0.625636 | newly 5 weeks spec | 17. | 3.08 | {0., 7.} | 81.8824 | 0.975505 | newly 5 weeks cumul | 130. | 34.08 | {13., 69.} | 75.5282 | 0.691254 | newly 54 weeks cumul | 176. | 41.6267 | {15., 84.} | 77.303 | 0.732728 |
